# Protein-Coding Genes’ Retrocopies and Their Functions

**DOI:** 10.3390/v9040080

**Published:** 2017-04-13

**Authors:** Magdalena Regina Kubiak, Izabela Makałowska

**Affiliations:** Department of Integrative Genomics, Institute of Anthropology, Faculty of Biology, Adam Mickiewicz University in Poznan, 61-614 Poznan, Poland; magdalena.kubiak@amu.edu.pl

**Keywords:** retrotransposon, retrotransposition, retrocopy, retrogene, gene duplication, genome evolution

## Abstract

Transposable elements, often considered to be not important for survival, significantly contribute to the evolution of transcriptomes, promoters, and proteomes. Reverse transcriptase, encoded by some transposable elements, can be used in *trans* to produce a DNA copy of any RNA molecule in the cell. The retrotransposition of protein-coding genes requires the presence of reverse transcriptase, which could be delivered by either non-long terminal repeat (non-LTR) or LTR transposons. The majority of these copies are in a state of “relaxed” selection and remain “dormant” because they are lacking regulatory regions; however, many become functional. In the course of evolution, they may undergo subfunctionalization, neofunctionalization, or replace their progenitors. Functional retrocopies (retrogenes) can encode proteins, novel or similar to those encoded by their progenitors, can be used as alternative exons or create chimeric transcripts, and can also be involved in transcriptional interference and participate in the epigenetic regulation of parental gene expression. They can also act in *trans* as natural antisense transcripts, microRNA (miRNA) sponges, or a source of various small RNAs. Moreover, many retrocopies of protein-coding genes are linked to human diseases, especially various types of cancer.

## 1. Introduction

A large fraction of human and other eukaryotic genomes consist of sequences that originated, directly or indirectly, as a result of transposable elements (TE) activities. Most of these genomic elements are considered to be nonessential to survival. However, TEs have a significant influence on genome evolution. TEs are probably most commonly known as recombination hotspots; however, they also contribute to the evolution of promoters and proteomes. Considering the direct contribution to proteomes, two scenarios exist: the coding potential of a TE is “domesticated” to perform host cellular function or TE-derived sequences are exapted into a coding portion of existing genes to generate novel protein variants [1]. One of the most impressive examples of a domesticated TE is the recombination-activating protein RAG1 [2]. This protein was derived from the transposase gene of a *Transib* DNA transposon 500 million years ago [3]. Another great example is the domestication of the *gag* gene. As many as 85 *gag*-like genes might exist in the human genome [4]. The second direct contribution to proteomes is exaptation, i.e., cooptation of different TE fragments to a new role. We call exapted those TE fragments that became a part of a coding sequence (CDS) but do not code for a protein domain attributed to their original function. In humans *Alu* sequences are major donators of exons, but exons acquired from other elements, such as LINEs, endogenous retroviruses, and DNA transposons, have also been reported [5]. Examples of the exaptation of an endogenous retrovirus envelope (*env*) gene are the primate genes *Syncytin-1* and *Syncytin-2*, which might be involved in the formation of the placenta [6].

Reverse transcriptase (RT), encoded by some TEs, can be used in *trans* to produce a DNA copy of any RNA molecule in the cell. This copy, reintegrated into the genome, will most likely be “dead on arrival” because none of the regulatory elements can be copied in RNA-mediated gene duplication. Therefore, these sequences are often called retropseudogenes or processed pseudogenes. Although the majority of these retrocopies are in a state of “relaxed” selection and remain “dormant” because they are lacking regulatory regions, many become functional. The evolutionary path of these functional retrocopies, called retrogenes, is not uniform. In the course of evolution, they may undergo subfunctionalization and share their function with their parent [7], develop a brand new function (neofunctionalization) [7], or replace their progenitors [8].

Retrogenes were long considered to be unimportant copies, but are currently called “seeds of evolution” since they have made a significant contribution to molecular evolution [9]. It has been shown that retrogenes play an important role in the diversification of transcriptomes and proteomes and may be responsible for a wealth of species-specific features. Some of these differences are highly important in medical research and may be the reason why results from animal studies cannot be transferred to humans. For example, the functional mouse retrogene *Rps23r1* reduces Alzheimer’s beta-amyloid levels and tau phosphorylation [10]. This particular retrogene is rodent-specific and does not exist in the human genome. Another elegant example of the functional phenotypic effect of retroposition was demonstrated by *fgf4* retrogene studies. Insertion of this retrogene is responsible for chondrodysplasia in dogs. All breeds with short legs are carriers of the *fgf4* retrogene [11].

The discovery that retro sequences considered “junk DNA” may be functional and play a crucial role in shaping genome-specific features was one of the most surprising breakthroughs of human and other genome analyses. A large number of studies were recently performed to explore these unique sequences, yet our knowledge of retrogenes’ evolution is exceptionally limited. In this review paper, we present recent studies aiming to decipher the functions of transcriptionally active retrocopies of protein-coding genes.

## 2. Retrotransposons as a Source of Cellular Reverse Transcriptase

**Figure 1:**
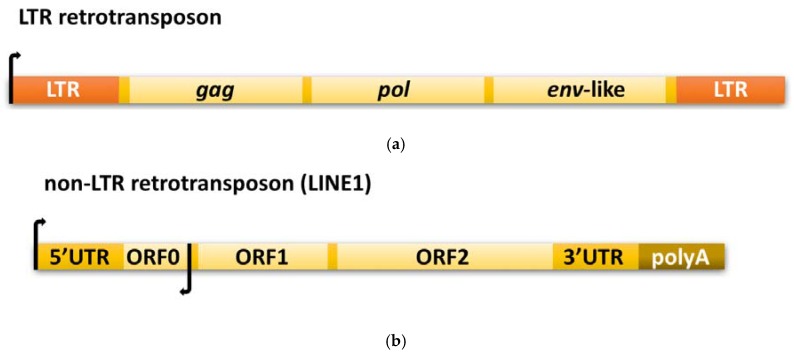
Schematic representation of the structural organization of (**a**) long terminal repeat (LTR) and (**b**) non-LTR retrotransposons. A detailed description can be found in Section 2.2 and Section 2.3.

The possibility of the reverse flow of genetic information from RNA to DNA was initially proposed in research conducted on the chicken Rous sarcoma virus [12]. The suggestion that the viral RNA genome can be transcribed into a DNA sequence and integrated into the host genome, together with the subsequent discovery of adequate enzymes [13,14], received the Nobel Prize in 1975. At that time, various mobile genomic elements, such as *Ty* in yeast [15] and LINE1 in human [16], were found to encode a reverse transcriptase, which was quickly associated with their mobilization abilities. This abundant group of “jumping genes”, called retrotransposons, has been divided into two families characterized by the presence or absence of flanking long terminal repeats (LTRs) (Figure 1). The first group includes retroviral-like elements with LTRs, and the second consists mainly of long interspersed nuclear elements (LINEs) and short interspersed nuclear elements (SINEs) without LTRs. The LTR and non-LTR retrotransposons can be further subdivided into autonomous retrotransposons, which encode proteins required for mobilization, and nonautonomous retrotransposons, which utilize the retrotransposition machinery of the others.

The origin of retroelements and the evolutionary relation between them and retroviruses are not currently clear. The major hindrance in evolutionary analysis is the lack of unitary molecular characteristics across all retrosequences. Despite this limitation, several approaches have been used to portray the relations, for example, comparison of the reverse transcriptase [17] or ribonuclease H domains [18]. According to these analyses, non-LTR retrotransposons are the oldest group of retroelements and might be derived from various sequences, for example, having a common ancestor with RNA viruses [17] or originating from the prokaryotic group of II introns, also called retrointrons [18,19]. In the case of LTR retrotransposons, the evolutionary path is even more ambiguous. Doubtless, they are closely related with retroviruses, which is reflected in the common structural traits and notable similarities during the retrotransposition process. Autonomous LTR retrotransposons contain *gag* and *pol* genes encoding structural proteins required for the formation of the virus-like particles and enzymes involved in reverse transcription and incorporation of new copies into the genome. The lack of the envelope (*env*) gene, which enables recognition and infection of the host cell, is considered a main difference between retrotransposons and retroviruses. Although *env*-like genes have been found in some retrotransposons, their function is not fully understood [20,21]. Due to these facts, it was suggested that retrotransposons evolved from retroviruses that lost their infectious properties as a result of mutational inactivation of the genes responsible for intercellular movement. Alternatively, LTR retrotransposons could have arisen from ancestral non-LTR retrotransposable elements, with acquisition of the *env* gene providing an opportunity for retrovirus evolution [17,18,19].

**Figure 2:**
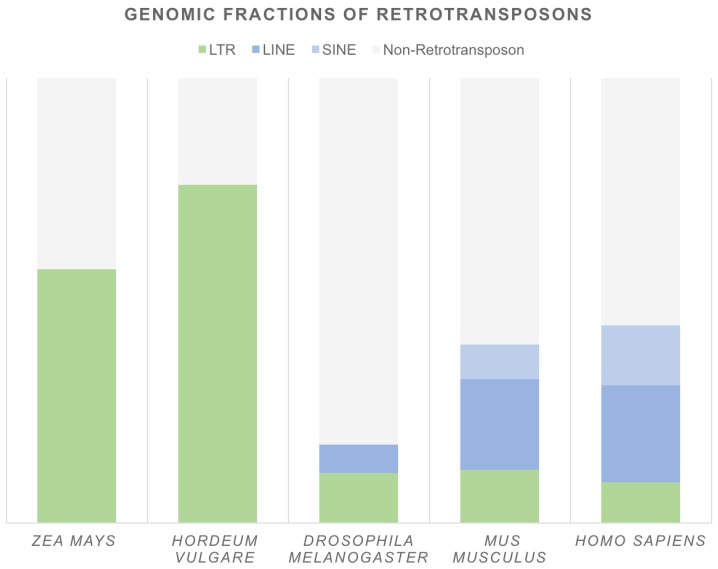
Genomic fractions of retrotransposons in selected genomes. Based on: *Zea mays* [23], *Hordeum vulgare* [25], *Drosophila melanogaster*, *Mus musculus* (genome version mm10), *Homo sapiens* (genome version hg38)—Repeat Masker online dataset [28].

Both LTR and non-LTR retrotransposons are abundant in eukaryotic genomes (Figure 2). In plants, the most prominent fraction is composed of LTR retrotransposons and is frequently associated with an enlarged genome size. A good example is *Zea mays*, in which insertions of retrotransposons have doubled the genome size during the past three million years of evolution [22]. LTR retrotransposons occupy at least 55% of the *Z. mays* and 76% of the *Hordeum vulgare* genomes [23,24,25] and compose up to 58% and 91% of the *Allium cepa* and *Asparagus officinalis* DNA contigs, respectively [26]. Non-LTR retrotransposons are less abundant and usually cover only a few percent of the whole genome. Nevertheless, analysis of 23 plant genomes indicated that structurally intact (and therefore potentially active) LINEs are present from model species, such as *Arabidopsis thaliana*, to very complex genomes, such as *Picea abies* [27]. In metazoa, compared to in plants, retrotransposons usually constitute a smaller genome fraction (Figure 2). A remarkable example is one of the model organisms, *Drosophila melanogaster*, in which retrotransposons compose approximately 17% of the genome. In mammals, non-LTR retrotransposons are the most common; they represent approximately 28% and 35% of the mouse and human genomes, respectively. In contrast, LTR elements compose only 12% and 9% of these genomes [28]. However, not all retrotransposon families are still active. For example, only three non-LTR retrotransposon families—long interspersed elements 1 (L1s), *Alu* elements, and SVAs (named after their composite parts: SINE-R, VNTR (variable number of tandem repeats), and an *Alu*-like sequence)—are actively mobilized in the human genome [29].

### 2.1. Retrotransposition of Nonautonomous Retrotransposons and Gene Copies

While autonomous retrotransposons encode their own mobilization machinery, the generation of nonautonomous retrotransposons and retrotransposed gene copies has long remained unclear. In the early 1980s, the analysis of repetitive sequences originating from small nuclear RNA and 7SL RNA showed that some are flanked by short direct repeats [30]. Because analogous repeats were found in combination with endogenous retroviruses and other mobile elements, a similar mechanism involving the insertion of reverse-transcribed RNA into the genome was suggested.

The endogenous reverse transcriptase activity and its ability to retrotranspose cellular non-retrotransposon mRNA were tested in a minigene system in HeLa cells [31]. Copies of a reporter gene with all characteristics indicating RNA-dependent duplication, including lack of introns, the presence of a polyA tract, and short repeats flanking the sequence, were found. Furthermore, the experiment showed that retrotransposition of mRNA in mammalian cells is still an active process. Further experimentation with mouse and human cells transfected by vectors containing reverse transcriptase revealed functional differences between the enzymes from retroviruses and those from LINE retrotransposon [32]. These results demonstrated that, in contrast to retroviral reverse transcriptase, LINE RT could generate reverse transcripts from RNAs, which do not show any sequence specificity or similarity to the LINE themselves. Therefore, autonomous non-LTR retrotransposons might be the source of endogenous RT involved in the formation of other retrotransposed genetic elements. Experiments performed by the same group [33] confirmed that LINE1 can act in *trans* and give rise to new retroposed gene copies. However, LINE1 elements are more effective in *cis*, which could be a consequence of the close proximity of LINE1-derived mRNA and proteins during translation or the limited life-time of protein in the lack of LINE1 mRNA [34]. This conclusion also explains the abundance of LINE1 elements in the genome compared to retrotransposed genes, as it was demonstrated that no more than 0.05% of the retrotransposition events conducted by LINE1 are related to retrotransposed gene formation [34].

Because the majority of experiments were conducted on animals, mostly mammalian models, the knowledge of retrotransposition in plants is restricted. Moreover, LINE elements constitute only a small fraction of the plant genome; their retrotransposition and creation of new gene copies is limited, but possible [35]. Since the first plant retrocopy was reported in *Solanum tuberosum* in 1987 [36], many retrotransposed genes have been found in various plant species [37,38,39,40]. For instance, a recent analysis of *A. thaliana* identified 251 retrocopies, of which 216 were described as novel [40]. In the RetrogeneDB2 repository, 1821 retrocopies in 37 plant species were identified [41]. Interestingly, analysis of plant retrocopies showed that LTR retrotransposons might also be involved in retrocopy formation. One of the best documented cases is the *Bs1* retrotransposon, which participated in the creation of retrocopies of three different cellular genes in the *Z. mays* genome [42,43]. Moreover, several additional retrocopies showing the signatures of LTR-mediated retroposition were recently found in both invertebrates and vertebrates [44]. Tan and coworkers [44] showed that LINE1-linked retrotransposition is dominant in mammals, whereas in mosquito, zebrafish, and chicken, retrocopies are created by both LTR- and non-LTR–mediated mechanisms. All polymorphic retrocopies found in *Drosophila* were formed by LTR retroposition. This type of retrotransposition is also more common in plants. Recent analysis of retrocopies derived from circular RNA (circRNA) in the mouse genome has shown LTR sequences localized in the flanking regions, which may suggest LTR-mediated retrotransposition [45].

Although the majority of retrotransposons have lost the capacity for mobilization—for instance, no more than 100 L1 in the human genome encode functional retrotranspositional machinery [46]—they are found to play various roles. They can shape genomes by acting as recombination hotspots or participating in exon-shuffling. They may also be used as new regulatory elements and alter the chromatin structure, thus influencing neighboring gene expression [47]. However, due to the integration of retrotransposons in random sites and the high likelihood of deleterious effects of insertion [48], different mechanisms of retrotransposition regulation evolved. Multilevel pathways are present in cells, starting from epigenetic silencing of retrotransposons by genomic DNA methylation and histone modification [49] to post-transcriptional positive and negative regulation [50].

### 2.2. LTR Retrotransposon-Based Transposition

The autonomous LTR retrotransposons are a diverse group; however, some common traits exist. They usually span several kilobases, are flanked by long terminal repeats, and contain promoters for RNA polymerase II localized in LTRs; however, only one RNA Pol II transcribes the retroelement. They encode at least two genes, which can overlap, be separated by terminal codons, or be fused into a single open reading frame [21,51]. One of the genes, *pol*, encodes various enzymatic domains, including reverse transcriptase and integrase, while the second, *gag*, produces structural proteins involved in the formation of virus-like particles (VLPs) (Figure 1a).

**Figure 3:**
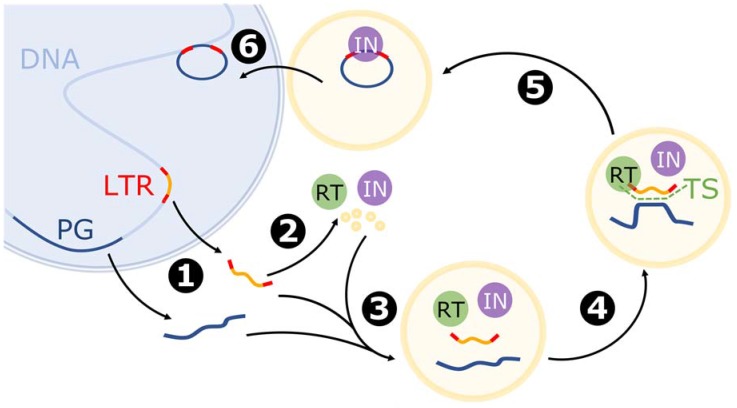
Model of mRNA retrotransposition mediated by the LTR retrotransposon (based on [20,44,51]). The blue sphere represents the nucleus. Stages: 1: Transcription of the LTR retrotransposon (LTR) and parental gene (PG). 2: Translation of LTR-encoded proteins, including reverse transcriptase (RT), integrase (IN), and proteins building virus-like particles (VLPs). 3: Formation of VLP with LTR-derived mRNA and parental gene mRNA. 4: Reverse transcription and template switch (TS). 5: Formation of the cDNA-IN complex. 6: Translocation and integration of chimeric cDNA into the genome.

The mechanism of retrotransposition is similar to that observed in retroviruses. The process begins with transcription of the LTR retrotransposon by RNA polymerase II, after which the newly synthesized RNA is transported to the cytoplasm [20,51]. Next, translation and formation of VLPs occurs. Reverse transcriptase, integrase, and RNA molecules are typically packed in VLPs, and by chance cellular mRNA may also be encapsulated [20,44]. Reverse transcription usually starts by annealing tRNA to a primer binding site near the 5′ LTR. The microsimilarities between the LTR retrotransposon and cellular mRNA allow for template switching between these two molecules and therefore for incorporation of an mRNA sequence into the emerging cDNA [44]. Moreover, stretches of microsimilarity can appear in multiple places along the gene. Theoretically, template switching can occur several times, but frequently only a limited part of the parental mRNA is reverse transcribed. Furthermore, second strand synthesis occurs from a polypurine tract near the 3′ LTR. Next, the LTR ends of the new cDNA are bound by integrase (IN) and the VLP localizes a nearby nucleus. Finally, the cDNA–IN complex is transported to the nuclei, and integrase cuts the cellular DNA and joins the released ends with LTRs from the retrotransposed copy [51]. The complete cDNA is finally integrated into the genome, generally at a random site (Figure 3). In contrast to LINE1-mediated retroposition, new retrocopies acquire long tandem repeats and thus a promoter sequence, which enables further transcription and retrotransposition [44].

### 2.3. Non-LTR Retrotransposition Mechanism

The most abundant non-LTR retrotransposon in mammals is LINE1 (L1); however, the set of the elements is quite variable between individuals [52]. For instance, a new insertion of L1 in humans occurs in between 1/95 and 1/270 of newborns [53]. Intact L1s are up to 6 kb in length and contain an internal promoter with sense and antisense activity localized in the 5′ untranslated region [54]. We can distinguish two open reading frames: one encoding a smaller RNA-binding protein with chaperone activity [55] and the other encoding a larger protein with endonuclease and reverse transcriptase domains. Mutational analysis of these two open reading frames showed that both proteins are required for retrotransposition [56]. Additionally, a third, primate-specific open reading frame was recently found on the antisense strand [57], but the biological function of the transcript is not clear. The majority of L1 elements also contain a polyA tract in their 3′ region (Figure 1b).

**Figure 4:**
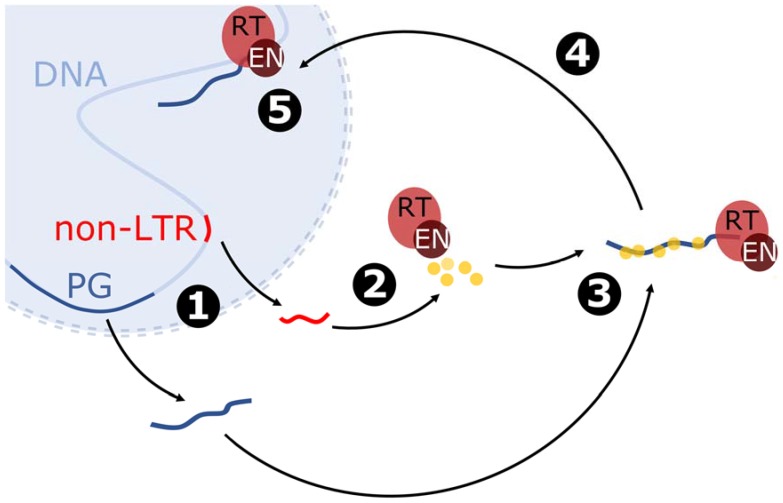
Model of mRNA retrotransposition mediated by non-LTR retrotransposon referring to LINE1. The blue sphere represents the nucleus; the dotted line shows the probability of nucleus membrane disruption. Description of the stages: 1: Transcription of the non-LTR retrotransposon (non-LTR) and parental gene (PG). 2: Translation of non-LTR–encoded proteins, including proteins with reverse transcriptase (RT) and endonuclease (EN) domains and RNA-binding proteins with chaperone activity. 3: Formation of ribonucleoprotein particle by binding of the proteins to the polyA tail of the parental gene mRNA. 4: Transport of ribonucleoprotein particle near the genome. 5: Reverse transcription and incorporation of the parental gene.

As experiments have shown, the LINE1 element can be transcribed by RNA polymerase II to mRNA, which is further used in translation and reverse transcription processes (Figure 4) [31,56]. The L1 mRNA is transported to the cytoplasm and translated, and the newly synthesized proteins interact with RNA in such a way that the one with enzymatic domains binds the 3′ end, while the smaller ones with chaperone activity are attached along the entire RNA molecule [58]. The spatial distribution and proximity may influence the *cis* preference of L1 retrotransposition. However, small chaperone proteins encoded by L1 may also bind to other RNA molecules. For example, LINE1s are responsible for the *Alu* (from 7SL RNA) [59], snRNA [60] and mRNA retrocopies [33]. LINE1s can also mobilize primate-specific SVA retrotransposons [61,62], hY RNA [63] or even human endogenous retroviruses [64]. However, the observation that removal of the polyadenylation signal results in a loss of retrotransposition [65] indicates that the presence of a polyA tail may be one of the requirements for mobilizing RNA via a LINE1-derived mechanism.

The ribonucleoprotein particle formed by RNA and LINE1-encoded proteins needs to get close to the chromosome DNA, where the target-primed reverse transcription occurs. However, it is not clear how the import to the nucleus happens. It was proposed that retrotransposition occurs during cell division when the nuclear membrane is disrupted [66]. However, there is also evidence for retrotransposition in non-dividing cells [67,68] and, therefore, cell division may not be necessary for the ribonucleoprotein transport near genomic sequence. Nevertheless, the reverse transcriptase domain begins synthesis of a new DNA strand on an RNA template using the free 3′-OH resulting from endonuclease cleavage of the genomic sequence [69]. Alternatively, non-classic L1 insertion into pre-existing gaps in DNA can occur [70]. The subsequent steps have not been thoroughly studied, but it was proposed that a second nick is generated downstream, thereby enabling second DNA strand synthesis. The final step may also include creating target site duplication (TDS) of variable length. Additionally, in this process, the 5′ site of the newly arisen copy is often truncated.

## 3. Number of Retrocopies across Genomes

The identification of retrocopies has been the subject of many studies and with the increasing number of sequenced genomes, as well as data from high-throughput experiments, new retrocopies are being discovered in various organisms. Examples of recent analyses include the comparative genomic study of the green algae retrogene repertoire [71] and inter-specific segregating retrocopies in cynomolgus and rhesus monkeys [72].

Basic annotations for retrotransposed gene copies, containing the localization and identified parental gene, are incorporated into several online databases. However, the most frequent description of these sequences is “processed pseudogene” or just “pseudogene”, which could be misleading as it does not directly indicate origin by retrotransposition. The major difficulty in annotation is the lack of specific sequence motifs and the high rate of mutation accumulation in retrocopies. Even the most obvious feature, a lack of introns, does not have to be preserved, as retrogenes are known for intron incorporation [73]. Moreover, they may also acquire novel exons or be fused with another gene and used as an alternative exon [74]. On the other hand, intronless genes are not always an outcome of a retroposition event. For instance, single-exon histone-encoding genes are believed to have originated in prokaryota [75]. Other retrocopy traits, such as the polyA tails and insertion site repeats, are found only in evolutionarily young retrocopies. Regardless, the best approach to retrocopy identification so far is based on alignment of known protein-coding sequences to the genome.

The Ensembl genome browser is one of the major resources of publicly available genomic information. In addition to datasets analyzed in an automated way, manually curated data from the HAVANA project are included [76]. Annotation of retrocopies, called “processed pseudogenes”, is based on an imperfect alignment between the genome and protein sequences, which enables multi-exon and single-exon gene models to be obtained. These single-exon annotations are interpreted as intronless, and therefore possibly retrotransposed, gene copies [77]. A similar method was applied in the PseudoPipe pipeline to predict all types of pseudogenes in the eukaryotic genomes stored on the Pseudogene.org server [78]. A slightly different approach was applied in UCSC Genome Browser, which besides Ensembl is the largest collection of genomic annotations. Here, the method for retrocopy identification was based on the RetroFinder pipeline, in which mRNA sequences were aligned to the genome [74,79]. Similar to the previous approaches, multi-exon and single-exon hits were obtained. The intronless candidates for retrocopies were then passed through multistage feature-based selection. For instance, a number of ancestral genes and putative retrocopy exons, as well as the presence and position of the polyA tail, were considered. PseudoPipe and RetroFinder, together with the HAVANA annotations, were used to produce a high-confidence dataset of pseudogenes, including retrocopies, in the human and mouse reference genomes in the GENCODE project [80].

More conservative approaches for retrotransposed gene copy annotation were applied in retroduplication-dedicated databases. The main purpose of utilizing more restrictive prediction criteria is the minimization of false positive results. On the other hand, they also provide expanded information enriched by potential function, interspecies conservation, and expression studies. There are three retrocopy-specific databases available. The first, HOPPSIGEN, is focused on human and mouse genomes [81]. A wider range of retrocopies, identified in six primate genomes, is available in RCPedia [82]. In addition, RetrogeneDB2 stores retrocopy information for 62 animal and 37 plant species [41]. Moreover, this database includes expression validation based not only on RNA-seq experiments but also on expressed sequence tag (EST), transcription start sites (TSS), and chromatin immunoprecipitation sequencing (ChIP-Seq) data. Another distinguishing feature of RetrogeneDB2 is the inclusion of data from retrocopy number variation studies that show retrocopies’ indel frequencies across human populations [83].

The numbers of predicted retroposed pseudogenes across databases are summarized in Table 1. Although all the described methods rely on the alignment of known multi-exon coding genes (in the form of nucleotide or amino acid sequences) to the genome, the number of retrocopies differs because of the distinct filtering strategies, as well as the applied tools and parameters. For instance, the HOPPSIGEN dataset was obtained using the BLAST alignment tool [84], while in RetrogeneDB2, database retrocopies were identified on the basis of sequence alignments generated by LAST [85] (Table 2). Additional information about putative retrogenes can also be acquired from intronless gene databases. For instance, the IGD database [86] and SinEx DB [87] contain sets of single-exon coding genes for human and 10 mammalian genomes, respectively.

## 4. Molecular Functions of Genes Retrocopies

In the retroposition process, the parental regulatory elements are usually not inherited, and the new copy often slowly decays and is silenced by the accumulation of degenerative mutations in a process called pseudogenization or nonfunctionalization. However, a large number of transcripts originating from retrocopies were found in cells, which suggests the acquisition of active promoters. Retrogenes may use regulatory machinery of nearby genes or utilize distant CpG-rich sequences [88] and occasionally parts of their own sequence [89] to promote transcription. For instance, when the parental gene has multiple transcription start sites and the one located upstream of the promoter region is used, the retrotransposed transcript may contain a prominent part of the core promoter. A good example of such a case is the *PABP3* retrogene. Analysis of the sequence similarity between the abovementioned retrocopy and the parental gene showed high conservation of the 5′ upstream region, suggesting that the retrogene arose from a gene transcript containing a fragment of the promoter [89].

After gene duplication, the genome contains two similar genes, which may potentially have the same function. This initial functional redundancy, depending on the deleterious or beneficial impact on the organism, may be eliminated or preserved during evolution. Signatures of purifying selection, like intact open reading frames or lower rates of non-synonymous to synonymous mutation, are frequently used as evidence to support the putative functionality of the new copy [7]. However, sequence conservation is not direct evidence of functionality. Experiments focused on the molecular characteristics of retrogene products are usually necessary to confirm expression and to assess the function of the analyzed molecule.

**Figure 5:**
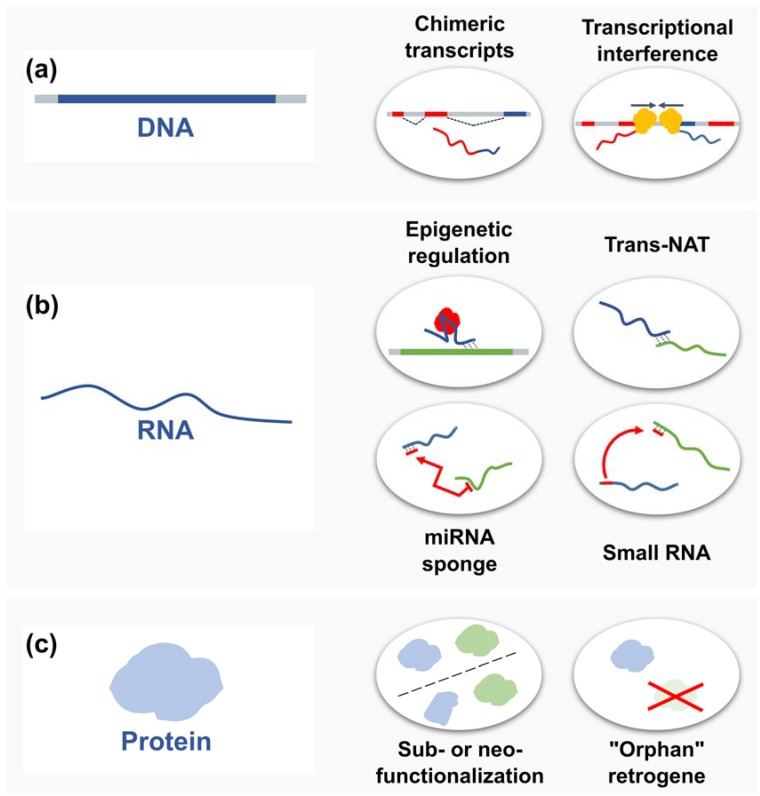
Selected functions of retrogenes. (**a**) On the DNA level, for example, retrogenes may be used as alternative exons and create chimeric transcripts or be involved in transcriptional interference; (**b**) as RNAs, they can participate in epigenetic regulation of parental gene expression and act as trans-natural antisense transcripts, microRNA (miRNA) sponges or a source of various small RNAs; (**c**) retrogenes can also encode proteins, which might retain the parental gene function (subfunctionalization), evolve a new function (neofunctionalization), or even functionally replace the parental gene (“orphan” retrogenes).

The development of high-throughput sequencing methods has enabled wide characterization of genomes, transcriptomes, and even epigenomes of various organisms, as well as particular organs and tissues. Sequencing experiments provide information about gene expression, methylation patterns, and DNA–protein interactions, which may be used to create a complex description of retrogene functionality and regulation. However, the analysis of short reads produced by next-generation sequencers is challenging because of the difficulty in assigning them to one of two or more highly similar sequences, such as the parental gene and its retrocopies. Nevertheless, new technologies that produce long reads from a single molecule have improved, and retrocopy expression analyses should eventually become less problematic. While analytical problems may always exist, our current knowledge about retrocopies and their function is quite extensive, and many different examples are well documented (Figure 5).

### 4.1. Protein-Coding Retrogenes

Retention of highly similar expressed sequences is often disadvantageous; therefore, conservation of the same gene function in its retrocopy is rare. Zhang [90] suggested that duplicates could be possible only in cases of highly demanded genes, such as rRNAs and histones. Although sharing the same function appears to be a natural consequence of gene duplication, retrogenes are often regulated in a different way than their ancestor genes because of distinct regulatory mechanisms. The main consequence of differences in regulatory machinery is spatio-temporal division of expression. In the most popular duplication-degeneration-complementation (DDC) model, the parental gene and retrocopy subdivide the ancestral function [91]. This mode of retrocopy evolution is called subfunctionalization.

One interesting but complex example of retrogene evolution is illustrated by a pair of retrocopies in *A. thaliana* [92]. The parental gene *CYP98A3* encodes the meta-hydroxylase engaged in the plant phenolic pathway and lignin biosynthesis. Two retrocopies of this gene, *CYP98A8* and *CYP98A9*, encode similar enzymes, which specialize in 3′- and 5′-hydroxylation of derivatives of spermidine localized in the pollen coat and wall. Analysis of the evolutionary history of the *CYP98* family shows that in *Brassicaceae*, the parental gene *CYP98A3* was retrotransposed and the *CYP98A8/9* ancestor retrogene went through tandem duplication [92]. Through further evolution, the loss of one copy was observed in some lineages; therefore, the remaining copy, *CYP98A8*, preserved the 3′- and 5′-hydroxylase activity. However, in the *A. thaliana* lineage, two copies were conserved, and subdivision of function occurred. *CYP98A9* acts as the 3′-hydroxylase, while *CYP98A8* acts as the 5′-hydroxylase. Moreover, the authors suggest that CYP89A9 retroprotein may have developed an additional function and play a role in flavonoid metabolism [92].

An example of subfunctionalization in humans is the cell cycle gene *CDC14B* and its retrocopy, *CDC14Bretro* [93]. Retrotransposition of the *CDC14Bpar* transcript occurred approximately 18–25 million years ago, and the ancestral function was probably conserved until the separation of African and Asian apes. However, in the African apes’ ancestor genome, several mutations in the 5′ end of retrogene sequence were fixed, and subcellular localization of the encoded protein was shifted from the microtubule to endoplasmic reticulum [93]. The authors suggest that the relocalization of the retrogene protein was due to a change in substrate and/or interaction partners and was related to the novel function development and specific testis/brain expression. Thus, this example may not be subfunctionalization but rather acquisition of a novel function that was previously not reported for the parental gene, i.e., neofunctionalization [94]. Another example where it is difficult to differentiate subfunctionalization from neofunctionalization is the *RAB6C* retrogene, which was retrotransposed approximately 21–25 million years ago in primates from the *RAB6A* gene [95]. In contrast to the parental gene, it is expressed in the centrosome, whereas the ancestor protein is found in the Golgi apparatus. This subcellular shift is probably a result of C-terminal extension impeding interactions with the Golgi. A *RAB6C* depletion experiment resulted in tetraploidization and duplication of the centrosome. Therefore, the retrogene-encoded protein developed a new function and is perhaps responsible for controlling cell cycle progression [95].

A retrogene-encoded protein may participate in an ancestral or new metabolic pathway, as shown above, but the process of retrocopy translation itself can also have an impact on the parental gene. An interesting example of this phenomenon is the connexin 43 (*Cx43*) gene and its retrocopy. Both encode proteins; however, retrogene expression is limited to breast cancer [96,97]. The *Cx43* retrogene has an intact open reading frame and encodes a protein of the same size as the parental gene, yet the ancestral function is not fully retained. While the parental *Cx43* gene is involved in cell growth control and intercellular communication, the retrogene seems to be not engaged in the second one [96]. Interestingly, further research indicated that the translational machinery preferentially binds to the retrogene, causing a shift in the parental gene mRNA from a polyribosome to monoribosome fraction, resulting in decreased expression of *Cx43* [97]. Silencing of the retrogene resulted in increased *Cx43* RNA and protein levels, supporting the regulatory role of the retrogene [97].

Another study of tumor-suppressor gene *TP53* suggested that gene duplicates, which arose via retrotransposition, play a role in the reduction of cancer risk in elephants [98]. Two of 19 retrogenes of the *TP53* gene, *TP53RTG12* and *TP53RTG19*, can be translated and enhance the DNA-damage response. In comparison to the parental TP53 protein, there are three significant differences: truncation in the DNA binding domain, lack of a nuclear localization signal, and lack of an oligomerization domain. However, the interaction motif, which enables binding to a negative regulator, is conserved. Therefore, the researchers suggested two putative models of function for this protein. The retrogene-encoded proteins may bind to and block a TP53 negative regulator or may directly bind to the parental gene protein and prevent its ubiquitination [98].

A retrogene may not only share a function with the parental gene but may become a functional replacement after pseudogenization or deletion of its progenitor. The first so-called “orphan” retrogenes were discovered as a result of a comparative analysis of worm, chicken, and human genes. All 25 such cases identified in the human genome represent known and well-studied genes that were not previously recognized as retrocopies. Moreover, seven of them are associated with various human diseases, including diabetes, attention-deficit/hyperactivity disorder, congestive heart failure, and Huntington’s disease. One of them, linked to hereditary spastic paraplegia, is the *CHMP1B* retrogene encoding chromatin-modifying protein 1B. The parental gene was pseudogenized in the ancestor of Old World and New World monkeys, but it is still active in rodents [8]. Another study identified a partially overlapping set of 10 “orphan” retrogenes [99]. Retroduplication and loss of parental genes was also found as a major mechanism involved in genome evolution and the generation of intronless genes in tunicates [100].

Another scenario of retrocopy function is dosage compensation when the level of the parental gene product is, for whatever reason, insufficient. For example, two testis-specific retrogenes, *RPL10L* and *RPL39L*, may compensate for their parental genes, which are inactivated during spermatogenesis [101]. A similar mechanism was proposed for the *HNRNP G-T* retrogene, which may functionally replace the parental protein in the course of meiosis [102].

Evidence of retrogene translation was also found during high-throughput data analysis. Because protein levels correlate with the levels of mRNA associated with polyribosomes, Mascarenhas and colleagues [103] analyzed the polyribosome loading of all RNA classes. An RNA sequencing experiment performed on cytosolic extracts and polyribosomal fractions showed that 18 pseudogenes exhibit significant polyribosome enrichment, which may suggest protein-coding potential [103]. Sixteen of these pseudogenes were found to be retrocopies.

Direct evidence of retrogene translation may be obtained via mass spectrometry. One interesting example of proteomic data utilization was an attempt to improve and further refine mouse genome annotations [104]. Analysis of 10.5 million tandem mass spectra enabled confirmation of the translation of known genes as well as identification of new protein-coding genes. Unique peptide hits were reported for nine retrocopies. One of them, retrotransposed from peptidylprolyl isomerase A (*PPIA*) gene, has two protein-coding variants that differ in the 5′ region. Intriguingly, none of these translated retrogenes have orthologs in the human genome [104]. Tandem mass spectra were also used in the proteomic profiling of 30 human adult and fetal tissues and primary hematopoietic cells [105]. In these studies, translation of more than 17,000 known protein-coding genes and 808 novel coding regions, including 140 pseudogenes, was confirmed. Although the authors did not discriminate between pseudogene subtypes, retrogenes can be identified in this dataset. For instance, nine peptide sequences were matched uniquely with the fibrillarin-like 1 (*FBLL1*) retrogene. Some of the identified pseudogenes, like MAGE family member B6 pseudogene 1 (*MAGEB6P1*), had common for retrocopies testes-specific expression, while others, like voltage-dependent anion channel 1 pseudogene 7 (*VDAC1P7*), had broad expression patterns [105].

### 4.2. Consequences of Retrogene Insertion for the Host and nearby Genes

Across the 84,483 retrogenes annotated in 62 animal species deposited in RetrogeneDB, approximately 20% (18,468) are inserted into the intron of another gene. In the case of the human genome, this proportion is even larger, and so-called nested retrocopies constitute 44% of the total. As mentioned previously, a retrocopy may use a promoter of the host gene to become transcriptionally active. Retrocopies are also frequently found in gene-rich and actively transcribed chromatin regions [88]. Depending on the position of the retrocopy, different destinies for the emerging transcripts are proposed. In the “hitchhike” scenario, a retrocopy inserted close to the 5′ end of the host gene uses a 5′ untranslated region (UTR) for its own transcription without a disruption of the host gene functions [7,88]. However, the production of resulting chimeric transcripts may theoretically reduce the normal host gene transcription level. Alternatively, a retrocopy insertion near the 3′ end of the host gene may produce a new exon for a new splice isoform [88].

The first reported chimeric transcript generated by retrocopy insertion into a previously existing gene was *jingwei* observed in *Drosophila* [106]. A decade later, a fusion gene was found in a vertebrate during a study focused on resistance to human immunodeficiency virus type 1 (HIV-1) [107]. Interestingly, retroposition of cyclophilin A (*PPIA*) into the *TRIM5* gene, which occurred after the divergence of New World and Old World monkeys, resulted in the origin of a novel protein that was probably able to attach ubiquitin to HIV-1 virion proteins [107]. However, chimeric transcript creation is not a widespread phenomenon across the human and mouse genomes. Baertsch and colleagues [74] analyzed 726 highly expressed retrocopies and identified only 34 cases as potential gene fusions. In another study, new chimeric transcripts of 13 human and 14 mouse retrocopies, together with the upstream exons of the host gene, were identified [99]. As an example, the authors present the mouse *Taf9* retrogene, which uses the first two exons of the *Ak6* gene to become active.

The retrogene or part of it may also be used as an alternatively spliced exon of the host gene or a new 3′ exon of a nearby gene. The *BRCA1* gene, for example, has an internal retrogene-derived exon, which generates a 22 amino acid cassette. In *SCP2*, *HLA-F*, and *KIAA0415*, alternatively spliced 3′ end exons arising from antisense retrocopy insertions were found [74]. Chimeric transcripts were also found in an analysis focused on human-specific retrocopies harboring 5′ CpG islands [108]. One is composed of exon 8 of the *RNF13* gene and *TMEM183A-r* retrogene and another is composed of the *HSF2BP* gene and *H2BFS* retrogene. In both cases the retrogene is located antisense to the parental gene orientation; therefore, these chimeric transcripts may potentially form RNA–RNA duplexes with their progenitors and participate in their regulation. The third chimeric transcript identified in these studies, created between the *VRK2* gene and *EIF3P3* retrogene, was expressed only in malignant prostate cancer cell lines [108]. Retrocopies expressed as a part of chimeric transcripts were also observed in plants. For instance, analysis of the retrogene repertoire in the genome of rice showed that more than one-third of the identified retrocopies recruit additional coding exons from nearby genes [38]. Retrogene-derived exons are found in many other plants’ proteins; for example, a polygalacturonase-inhibiting protein encoded by a chimeric gene acting against *Aspergillus niger* polygalacutonase [38,109].

As already mentioned, the origination of chimeric transcripts may influence the expression level of a host gene. However, this is not the only way in which retrocopies regulate the expression of other genes. A group of *cis*-regulatory transcription-related functions was proposed for neighboring genes [110]. First, two genes localized in the same genomic locus and transcribed from independent promoters may directly impede each other’s transcriptional processes [110]. This suppressive influence, called transcriptional interference, is a result of interactions between the dominant and sensitive promoters of overlapping genes. According to the promoter orientation and arrangement, several mechanisms of this process were proposed, including promoter competition, sitting duck interference, occlusion by another transient promoter occupation, collision of elongation complexes, and roadblocks precluding transcription [110]. Although transcriptional interference appears to be less frequent in higher eukaryotes, examples of this process have been reported; for instance, the results of an analysis focused on mouse and human genes that overlap in antisense orientation were consistent with the transcriptional collision model [111]. Transcriptional interference was also analyzed in the context of intronic retroelements and single-exon nested genes [112]. Using a minigene system and different deletion constructs, the *KTI12* retrogene located in the intron of the *TXNDC12* gene was analyzed. This particular case was selected based on the identification of three different ESTs, suggesting forced exonization of a portion of the *TXNDC12* intron upstream of the retrogene. The experimental results confirmed that expression of the *KTI12* retrocopy imposes utilization of cryptic acceptor splice sites and premature termination of *TXNDC12* gene transcription.

Host genes may also be affected by intronic retrogene methylation. Epigenetic regulation of expression is one of the mechanisms proposed for retrotransposon activity suppression; thus, a link between retrotransposition and methylation is strongly suggested. Recent analysis of retrocopy-associated CpG islands showed that 68% of them are methylated, which in comparison to the whole human genome is a significant proportion [113]. DNA methylation of cytosine bases at the CpG dinucleotides is a basic modification that occurs during genomic imprinting. Few examples of imprinted retrogenes have been reported. For instance, a systematic screen of known genes in mouse led to the identification of 11 imprinted retrogenes, of which three (*Mcts2*, *Dnajb3*, and *Oxct2a*) were nested in an intron of another gene. Interestingly, imprinting of these retrogenes is conserved in humans [114]. Detailed studies of the transcriptionally active *Mcts2* retrogene, inserted into the fourth intron of the *H13* gene, revealed that the retrogene’s promoter is silenced by methylation in the female germline [115]. Surprisingly, the choice of polyA signal by the host gene depends on this epigenetic promoter modification. It was shown that expression of the retrogene from the paternal allele forces utilization of the upstream polyA site, resulting in a truncated transcript of the host gene [115]. Similar observations were reported for the *Nap1 l5* retrogene and *Herc3* host gene [116] and other retrogenes (for review, see: [117]).

### 4.3. Retrocopy Impact on Parental DNA

The main implication for the coexistence of highly similar sequences in a genome is the possibility of direct exchange of DNA fragments in a homologous recombination event. Retrogenes, like other genomic duplicates, can participate in gene conversion. However, this process was shown to be less frequent for genes localized in distant genomic regions [118]; therefore, it is less likely to occur between retrocopy–parental gene pairs, as they are in most cases localized on different chromosomes [119]. The retrotransposition process is also proposed as a possible mechanism in the RNA-mediated intron loss observed in Eukaryota [120,121,122]. In this model, precise deletion of the parental gene intron occurs as a result of recombination between genomic DNA and spliced, reverse-transcribed mRNA. Intron loss analysis conducted on 684 groups of orthologous genes from seven eukaryotic species supported the proposed mechanism [123]. Another study suggested that higher reverse transcriptase activity is connected to higher frequencies of intron loss and larger numbers of retrocopies. A correlation between these events was observed in mammals [124].

Retrogenes may have an impact on parental genes by contributing to epigenetic regulation of their expression. Nuclear antisense transcripts can act as a scaffold for chromatin remodeling complexes and therefore guide them to genomic loci on the basis of sequence complementarity [125]. It was also demonstrated that retrogenes may participate in this mechanism. A well-known example is the tumor-suppressor gene *PTEN*, whose expression is regulated at multiple levels by the *PTENpg1* retrogene [126,127]. *PTENpg1* has three different transcripts, of which two are antisense to the parental gene. One isoform, called *PTENpg1 asRNA alpha*, recruits the DNA methyl transferase 3A to the parental promoter [127]. As a result, repression of parental gene expression occurs by the addition of three methyl groups to histone H3 Lys27.

### 4.4. Retrogene Regulatory Functions on RNA Level

#### 4.4.1. *Trans*-Natural Antisense Transcripts

The vast majority of transcribed retrogenes do not have conserved open reading frames and therefore have roles other than protein-coding functionality. Retrogenes may be expressed independently from both DNA strands, which increases the range of possible interactions on the RNA level. For instance, bioinformatic analysis of ESTs showed that retrogenes constitute 15% of the 87 pseudogenes that were found to be expressed from an antisense strand [128]. Moreover, recent studies focused on long non-coding RNAs that overlap retrocopies across the human genome identified three retrocopy-derived antisense RNAs (asRNAs). These retrocopies are potentially capable of forming RNA:RNA duplexes with their parental genes [129]. One of these long non-coding RNAs (lncRNAs) derived from the *HNRNPA1P7* retrogene may contribute to heterogeneous nuclear ribonucleoprotein A1 (*hnRNPA1*) pre-mRNA processing. The bioinformatic analysis strongly suggested that asRNA might mask the 5′ splice site in the sixth intron of parental gene transcript and therefore enable the expression of isoform with a longer sixth exon. Another identified lncRNA, antisense to a ribosomal protein L23a (*RPL23A*) retrocopy, is potentially able to mask microRNA target sites in seven splice forms of the parental gene, thus controlling their stability [129]. An interesting example was found in the snail *Lymnaea stagnalis*. A retrocopy of a gene encoding nitric oxide synthase (NOS) includes a region of antisense homology to its progenitor’s transcript. The antisense region of the pseudogene transcript forms an RNA:RNA duplex with the NOS-encoding mRNA and prevents its translation [130]. Antisense and sense transcripts can also interact. *PTENpg1*, described earlier in the context of epigenetic modification, has a second antisense transcript, *PTENpg1 asRNA beta*, which stabilizes the expression of the sense transcript [127].

#### 4.4.2. MicroRNA Sponges

Continuing with the example of *PTENpg1*, another gene regulation mechanism should be mentioned. The sense transcript of *PTENpg1* shares many microRNA binding sites with the parental gene and consequently exhibits “sponging” activity, manifested by releasing miRNA-mediated repression of *PTEN* [126]. *PTEN* was identified as a tumor-suppressor gene that negatively regulates the phosphatidylinositol 3-kinase signaling pathway involved in cell proliferation control [131]. Downregulation of *PTEN*, which occurs when the retrogene transcript is absent, enhances proliferation and cell growth and decreases sensitivity to cell death, which promotes tumorigenesis [126,131]. The *PTEN-PTENpg1* endogenous competition for shared microRNA molecules has an oncosuppressive effect on different human cancers, including prostate and colon cancers [126], melanoma [132], renal cell carcinoma [133], and hepatocellular carcinoma [134]. Since the identification of *PTENpg1* as a microRNA sponge and functional antisense RNA and its role in tumorigenesis, many retrogene-derived non-coding RNAs have been analyzed in this context [135,136,137,138].

One interesting example is the high mobility group A1 (*HMGA1*) gene and its two retrogenes, *HMGA1P6* and *HMGA1P7* [139]. The parental gene encodes proteins involved in chromatin architecture organization and therefore gene expression regulation. Whereas in adult normal cells *HMGA1* proteins are expressed at a very low level, they are overexpressed in cancers. Analysis of the retrogene–parental gene expression pattern in thyroid and ovarian carcinomas showed a positive correlation and suggested gene co-regulation [139]. Both investigated retrogenes conserved the miRNA target site of the parental gene. The abilities of miRNA–retrogene interactions were experimentally evaluated by transfection of miRNAs into human breast adenocarcinoma cells. A significant reduction in the *HMGA1*, *HMGA1P6*, and *HMGA1P7* mRNA levels was observed, which strongly supports the retrogene miRNA “sponging” activity. These retrogenes may also regulate the expression of other genes, including cancer-related ones, such as *HMGA2*, *VEGF*, and *EZH2* [139]. Another research group found that the *HMGA1P7* retrogene is also involved in the regulation of *H19* non-coding gene and *IGF2* gene expression in human breast cancer [140]. In contrast to the oncosuppressive function of *PTENpg1*, *HMGA1P6* and *HMGA1P7* show oncogenic activity and contribute to cancer progression.

Retrogenes may also compete with parental genes for other molecules. For instance, the *Cx43* retrogene transcript shows higher affinity to translational machinery than the parental gene mRNA and therefore decreases the parental protein level [97].

#### 4.4.3. Small RNA

Retrogenes have also been proposed as a source of several classes of small RNAs. For example, they may provide the sequence for novel miRNA genes. Primate-specific *miR-492* may be expressed from both *KRT19* gene and retrogene loci [141,142]. Interestingly, *hs-miR-492* was proposed to play a role in the progression of hepatoblastoma [142] and was found to be a proto-oncogenic miRNA, acting as a cell proliferation promoter in breast cancer [143]. Several other miRNAs stored in miRBase [144], the main source of miRNA annotations, lie within retrogenes. For instance, hsa-*miR-622*, which acts as a suppressor of tumorigenesis in cancers, including hepatocellular carcinoma [145], overlaps with the genomic locus of the *KRT18P27* retrocopy. Other examples include *hsa-miR-7161*, located in the *TATDN2P2* retrocopy, and *hsa-miR-4788*, overlapping *HMGB3P13*. However, experimental analysis must be conducted to verify the actual miRNA coding potential.

Piwi-interacting RNA (piRNA) involved in the formation of silencing complexes in animal germ lines can also be derived from retrocopies. Total small RNA profiling of the marmoset testis showed abundant expression of piRNAs [146]. Across various clusters, four antisense-oriented piRNAs localized within retrocopies were found, and the authors suggested that the piRNAs originating from these clusters might regulate the parental gene expression by cleaving mRNAs. Moreover, retrogene-derived piRNAs appear to be species-specific because clusters found in the marmoset were absent in the mouse [146]. Recent bioinformatic analysis focused on the small RNA of human sperm showed that piRNA clusters also contain non-coding genes, one of which overlaps with the *NPAP1P6* retrocopy [147]. Interestingly, recent evolutionary analysis showed that the parental gene of the *NPAP1P6* retrocopy, *NPAP1*, was created via duplication of a retrotransposed ancestral paralog derived from the vertebrate nucleoporin gene *POM121* [148]. *NPAP1* is a primate-specific imprinted gene that encodes a nuclear pore-associated protein associated with Prader–Willi syndrome. As the authors emphasized, this syndrome is linked with testis dysfunction, which supports the possible relation between many sperm piRNAs and the analyzed retrocopy [147]. Several additional piRNAs from retrocopies, including *IMPDH1P5*, *TMX2P1*, and *RP11-545A16.3*, which may potentially interact with the protein-coding genes due to high sequence complementarity, were identified [147]. Six retrogene-derived piRNAs that potentially regulate parental genes were found in late mouse spermatocytes [149]. For one of them, experimental evidence of post-transcriptional regulation of *Stambp* gene was provided. By generating two mouse strains with gene-trap insertions upstream of the retrocopy, the authors demonstrated relationships between the piRNA precursor, piRNA, and the parental gene levels, which clearly suggests a role of the retrocopy in *Stambp* regulation [149]. PiRNA clusters overlapping retrocopies were also found in other recent studies (e.g., [150,151]).

Antisense transcripts from retrocopies can pair with other transcripts, including those of the parental gene. This double-stranded RNA may be processed into endogenous small interfering RNA (endo-siRNA). Analysis of the small RNA profiles from wild-type and seven RNA-silencing mutants of *A. thaliana* showed overrepresentation of siRNAs in the transposons, retroelements, and pseudogenes, which may suggest that these sequences are regulated by siRNA-generating systems [152]. A year later, two scientific reports published in parallel showed that endo-siRNAs annotated in the retrocopy locus may regulate their parental genes’ expression in mouse oocytes [153,154]. For instance, 77 small RNAs were mapped to an expressed retrocopy (*Gm15681*) of the protein phosphatase 4 regulatory subunit 1 gene (*Ppp4r1*). Moreover, almost all were oriented antisense to the parental gene, which strongly suggested that the siRNAs originated from the retrocopy-parental gene double stranded RNA (dsRNA) region [154]. In another analysis focused on developing rice grains [155], among 145 pseudogenes identified as good candidates for generating antisense small RNAs, 16.6% were retrocopies. However, their *cis*-activity was questioned due to a low rate of identified gene–retrogene complementary regions from which siRNA can be produced [155]. A cluster of siRNAs derived from pseudogenes was also identified in African *Typanosoma brucei* [156]. More detailed studies were conducted for endo-siRNAs derived from human pseudogenes in hepatocellular carcinoma. A well-documented example is the human endo-siRNA from the retrogene of the protein phosphatase 1K (*PPM1K*) gene. The retrocopy can fold into a hairpin structure due to inverted repeats and can be processed in at least two endo-siRNAs, one of which downregulates the parental gene and NIMA-related Kinase 8 (*NEK8*) gene, inhibiting cell proliferation in hepatocellular carcinoma. Thus, the retrogene could be considered a tumor-suppressor gene [157].

Retrocopy-derived small RNAs were also found in the human transcriptome during characterization of the non-coding RNA repertoire. A higher density of small RNA was observed in retrocopies than in duplicated pseudogenes or coding genes. Interestingly, transcription-dependent H3K9me3 enrichment was observed in some cases, suggesting that pseudogene-derived small RNAs, including retrocopy-derived RNAs, may play a role in modulating the epigenetic suppression of those pseudogenes, as well as neighboring gene expression [158].

## 5. Retrogenes in Diseases

Many retrocopies of protein-coding genes are linked to human diseases, especially various types of cancer (for review, see: [159,160,161,162]). It was recently suggested that expression of evolutionarily young non-coding genes in tumors might be considered a new biological phenomenon [163]. A good example of a cancer-related retrogene is the *RHOB* gene, a tumor suppressor of the Rho GTPases family, which arose via retroposition in the early stage of vertebrate evolution [164]. Another retrogene, *UTP14c*, was linked to ovarian cancer predisposition [165]. An analysis of 293 samples representing 13 cancer and normal tissue types revealed 218 pseudogenes expressed only in cancer samples. Out of them, 178 were observed in multiple cancers and 40 were identified in a single cancer type only [166].

As many reports have shown, retrocopies can be used as diagnostic biomarkers, such as the *INTS6P1* retrogene, for which a low expression level in plasma is linked with hepatocellular carcinoma [167], or as prognostic markers, such as tumor-suppressive *PTENpg1* [126,132,133,134] and the oncogenic *HMGA1P6* and *HMGA1P7* [139,140] retrogenes mentioned in previous sections. Another retrocopy of the *HMGA1* gene is also linked with disease; its overexpression has been found in human type 2 diabetes. Further analysis indicated that this retrocopy post-transcriptionally regulates parental gene expression by competing for critical RNA stability factor and, as a result, suppresses the expression of the insulin receptor gene. Therefore, this retrocopy contributes to insulin resistance [168].

Due to the reactivation of retrotransposons in somatic cells during cancer development, the formation of new retrocopies was observed [169,170]. Somatically acquired retrocopies are present in lung and colorectal cancers. Moreover, insertions occur not only in intragenic regions but also in other genes, which may have implications for their expression. For instance, a *KRT6A* retrocopy replaced the 3′UTR of the *MLL* gene transcript and a *PTPN12* retrocopy caused deletion of the promoter and first exon of the *MGA* gene [170].

Retrogenes may also play active roles in the regulation of signaling pathways involved in inflammation [171]. A mouse retrocopy of ribosomal protein S15A gene, called *Lethe* (*Rps15a-ps4*), is induced by inflammatory cytokines TNFα and IL-1β. Moreover, it can bind and block RelA homodimers, which are required for NF-κB activation; therefore, *Lethe* plays the role of a negative inflammatory response regulator. Interestingly, the retrogene is expressed in an age-dependent manner [171]. Mutation in another retrogene, *TACSTD2* (tumor-associated calcium signal transducer 2) causes gelatinous drop-like corneal dystrophy, leading to blindness [172]. An insertion of a retrocopy, similar to insertion of *L1* or *Alu* elements, may disrupt a gene structure. An example of such event is insertion of a retrocopy of *TMF1* gene into the *CYBB* gene on the X chromosome. This insertion induced aberrant *CYBB* mRNA splicing and introduced a premature stop codon that resulted in chronic granulomatous disease [173].

Retrocopies were also incorporated into analyses conducted in the context of neurodegenerative disorders, including Alzheimer’s, Huntington’s, and Parkinson’s diseases [174], as well as muscular dystrophy [175].

As described above, the chimeric transcript resulting from cyclophilin A (*PPIA*) retrogene insertion into the *TRIM5* host gene in the owl monkey is involved in HIV-1 resistance [107]. Retrocopies may also be associated with the host response during pathogen infection in humans, as significant expression changes were observed after HIV-1 and human type 2 adenovirus infection [176,177]. For instance, expression pattern analysis after HIV-1 infection of human T-cells showed that the most upregulated pseudogene group was a group of retrocopies. Additionally, retrocopies accounted for eight out of 13 cases of underexpressed pseudogenes [176]. These results suggest that both tandemly duplicated and retroposed pseudogenes may be involved in host–pathogen interaction pathways.

## 6. Retroposition and Genetic Variation

Retroposition gives rise to considerable genetic variation between individuals. Recent developments in sequencing technology allow researchers to move beyond the analysis of individual genomes from model organisms to the study of retrocopies within a population. The 1000 Genomes Project [178] could be mentioned as an example of a large-scale sequencing project that enables the exploration of differences in copy-number variation within human populations. Recent studies [66,169,179], focused on the retrocopy repertoire in human populations, uncovered a total of 208 polymorphic retrocopies [180] called retroduplication variations (RDVs). Moreover, in two of them [66,169], RDV polymorphisms were used as genomic markers for the reconstruction of human population history. In another study, concentrated on retrocopies deletions, 214 indels that affected 190 retrocopies were identified. Out of them, 68 were found to be ancestral (i.e., their orthologs were found in at least one another Hominidae species) and the polymorphism of these retrocopies clearly resulted from a deletion. This study also showed a variation in the retrocopies’ expression level [83].

## 7. Conclusions

Retrocopies were long considered non-functional pseudogenes or even “junk DNA”; however, current studies show that they contribute significantly to molecular evolution. Retrocopies have been found as factors shaping differences between species, individuals, or even tissues and cell types; therefore, they are considered a source of genetic polymorphism. They are also important players in complex cellular pathways, including immune response and tumorigenesis. Moreover, a large and increasing range of putative retrocopy functions make them an interesting subject of molecular and medical studies. The numerous studies performed to date have enriched our comprehension of the course and dynamics of retrocopy–gene interactions at the DNA, RNA, and protein levels. Nevertheless, many questions remain unsolved, and further analyses are necessary to accurately describe the plant and animal retrocopy repertoire, evolution, and functions.

## Figures and Tables

**Table 1:** Comparison of retrocopy sets available in the described databases. In addition, the number of retrogenes annotated in the *Homo sapiens*, *Pan troglodytes*, *Macaca mulatta*, and *Mus musculus* genomes is shown.

Database	Plants	Animals	Number of Retrocopies			
			*Homo sapiens*	*Pan troglodytes*	*Macaca mulatta*	*Mus musculus*
Non-specific databases						
Ensembl ^1^	+	+	10,815	69	182	6999
UCSC ^2^	+	+	13,742	−	−	18,456
GENCODE ^3^	−	+	9074	−	−	6151
Pseudogene.org ^4^	+	+	8739	7505	−	9809
Retrogene-dedicated databases						
HOPPSIGEN	−	+	5206	−	−	3428
RCPedia	−	+	7831	7733	7544	−
RetrogeneDB2	+	+	4611	3285	2377	4148

^1^ Ensembl genome browser release 86 dataset filtered by “processed pseudogene” in BioMart; ^2^ UCSC RetroGenes v9 (*H. sapiens*) and v6 (*M. musculus*) dataset statistics; ^3^ GENECODE human (Release 25, GRCh38.p7), Mouse (Release M11, GRCm38.p4), “processed pseudogenes” with “gene” and “level 1” statuses were chosen from the comprehensive gene annotation GTF file; ^4^ class “processed” was selected for all organisms: chimp build 50 (CHIMP2), mouse build 84 (GRCm38), human build 83 (NCBI38).

**Table 2:** Approaches used in retrocopy-specific databases.

Database	Sequence Aligned to the Genome	Tool
HOPPSIGEN	gene coding sequence	TBLASTX
RCPedia	entire transcript	BLAT
RetrogeneDB2	protein	LAST
